# Future sea ice conditions and weather forecasts in the Arctic: Implications for Arctic shipping

**DOI:** 10.1007/s13280-017-0951-5

**Published:** 2017-10-27

**Authors:** Jean-Claude Gascard, Kathrin Riemann-Campe, Rüdiger Gerdes, Harald Schyberg, Roger Randriamampianina, Michael Karcher, Jinlun Zhang, Mehrad Rafizadeh

**Affiliations:** 1 University Pierre & Marie Curie, LOCEAN, 4 Place Jussieu, 75005 Paris, France; 2 Alfred-Wegener-Institut Helmholtz-Zentrum für Polar- und Meeresforschung, Bussestrasse 24, 27570 Bremerhaven, Germany; 3 Norwegian Meteorological Institute, P.O. Box 43, Blindern, 0313 Oslo, Norway; 4 Ocean Atmosphere Systems GmbH, Tewessteg 4, 20249 Hamburg, Germany; 5 Applied Physics Laboratory, Polar Science Center, University of Washington, Seattle, WA USA

**Keywords:** Climate change, Polar shipping, Sea ice, Weather forecast

## Abstract

The ability to forecast sea ice (both extent and thickness) and weather conditions are the major factors when it comes to safe marine transportation in the Arctic Ocean. This paper presents findings focusing on sea ice and weather prediction in the Arctic Ocean for navigation purposes, in particular along the Northeast Passage. Based on comparison with the observed sea ice concentrations for validation, the best performing Earth system models from the Intergovernmental Panel on Climate Change (IPCC) program (CMIP5—Coupled Model Intercomparison Project phase 5) were selected to provide ranges of potential future sea ice conditions. Our results showed that, despite a general tendency toward less sea ice cover in summer, internal variability will still be large and shipping along the Northeast Passage might still be hampered by sea ice blocking narrow passages. This will make sea ice forecasts on shorter time and space scales and Arctic weather prediction even more important.

**Electronic supplementary material:**

The online version of this article (doi:10.1007/s13280-017-0951-5) contains supplementary material, which is available to authorized users.

## Introduction

The Arctic climate is subject to a drastic change. A key element in Arctic climate change is the rapid decline in sea ice coverage and thickness seen in observations in particular since 2000. Research is needed to improve understanding of sea ice development and weather forecasting abilities since an increased human use of the Arctic in all kinds of economic sectors is anticipated. Here we report on results with respect to the potential future of large-scale sea ice development in the Arctic as they are relevant for shipping. We also make suggestions on how to improve weather forecasts, which are essential for regional sea ice forecasting on shorter time scales. The results described here also provide relevant input on the environmental conditions to studies of the potential development in other economic sectors such as oil and gas exploration and fisheries (see, e.g., Petrick et al. 2017).

In the EU-funded project ACCESS—Arctic Climate Change, Economy and Society, studies were performed to enhance the understanding of the interaction between this decline of sea ice and changes in the atmosphere and ocean. Long-term trends in response to anthropogenic forcing appear on the centennial time scale, but large natural variability of the atmosphere–ocean–cryosphere system is superimposed on time scales of years to a few decades, which hampers the detection of trends. Earth System Models are our only tools to predict the system, but they have their limits since they are sensitive to how well physical processes are represented and spatially resolved in the models and as a consequence of the natural variability.

In this paper, we discuss our abilities to hindcast and predict sea ice conditions in the Arctic Ocean. In the first part, we present new findings regarding the pan-Arctic sea ice evolution from 1980 to 2040. Earth system model simulations, chosen according to the models’ performance in comparison to observed sea ice concentration in the twentieth century, will be used to evaluate the evolution of the Arctic sea ice volume based on a subset of the Intergovernmental Panel on Climate Change (IPCC) program called Coupled Model Intercomparison Project phase 5 (CMIP5) (Taylor et al. 2012). From the past to present, we compare these simulations (Shu et al. 2015; Song 2016) with a model that assimilates observations [PIOMAS—Pan-Arctic Ice-Ocean Modelling and Assimilation System; Zhang and Rothrock [2003)] and sea ice volume estimations based on observed freezing conditions expressed as Freezing Degree Days (FDD) deduced from ERA-Interim reanalysis (Dee et al. 2011; Gao 2013) for the historical period. ERA stands for European reanalysis. ERA-Interim is a data set resulting from a global climate reanalysis covering the period from 1979 to the present. This combination allows a validation of the Earth system model results on the large spatial scale. We will then present results focusing on future sea ice conditions on a finer spatial scale, using the best performing CMIP5 model to drive a regional, finer scale model. In the second part, we zoom into the conditions in the shelf areas of the Arctic, as they are relevant for ship navigation, taking into account critical narrow passages along the Northeast Passage (NEP). In the third part, we will introduce recent findings regarding the options to improve high-latitude weather forecasts in the future, which is also pivotal for improving sea ice prediction in critical regions to allow safer operations in Arctic ocean regions.

## Pan-Arctic sea ice evolution

One of the main objectives of ACCESS concerned the future evolution of Arctic sea ice concentration and thickness, in particular in areas of potentially increased human activities in future, such as along shipping routes. However, the attempt to project this development into the future faces large uncertainties such as the actual development of future greenhouse gas emissions, the unavoidable uncertainties due to natural variability and errors of the Earth system models, our only tools to attempt a projection of the climate system development (e.g., Hawkins and Sutton 2009).

In the course of the activities of the IPCC process for the most recent report (CMIP5), more than 30 global Earth system models were used to project the development of the global climate, assuming a set of different scenarios for the potential future release of greenhouse gases (Taylor et al. 2012).^1^ For the Arctic regions, these models provide a large range of possible future environments, part of which may be due to model errors. This leads to the question of which of these simulations can be deemed the most realistic. A way to attempt at an answer is to subsample these model experiments to only make use of those that perform the best in comparison with observed historical data, in our case with sea ice observations. Since sea ice concentration derived from satellites provides relatively long, large-scale ‘observational’ record, it has been used here to select CMIP5 models. A detailed description of the selection process can be found in the Electronic supplementary material. In our studies, we make use of the four best performing model experiments to provide information on the possible range of sea ice extending for the next three decades 2010–2040 (for further information, see Riemann-Campe et al. 2014; Petrick et al. 2017). However, the spatial resolution of the Earth system models is too coarse for the purpose of evaluating near-coastal shipping routes such as the NEP (see supplementary material for additional information) and we need to derive information on finer spatial scales. Previous studies which analyzed sea ice along shipping routes based on CMIP5 model results derived projections on finer spatial scales by re-gridding the original coarser CMIP5 model grids (e.g., Laliberte et al. 2016; Khon et al. 2017) or they applied a statistical bias correction method to overcome the problematic coarse resolution (Melia et al. 2016). While such approaches have the advantage of allowing the analysis of a large number of experiments, they are unable to create information at spatial scales below the original CMIP5 model resolution, as for example the conditions in narrow straits. Here we apply a different approach using a ‘dynamical downscaling’: we use the atmospheric results from ensemble simulations of the best performing CMIP5 model, the Max Planck Institute for Meteorology’s Earth system model (MPI-ESM-LR; Notz et al. 2013), to drive a regional, coupled sea ice and ocean model with a higher spatial resolution for a downscaling experiment (DEXP8.5) covering a historic period and the upcoming decades (1950–2040).^2^ In contrast to the studies mentioned above, this approach offers the advantage of fully resolved physics on the finer grid, though at the price of much higher effort, reducing the number of possible experiments.

**Fig. 1:**
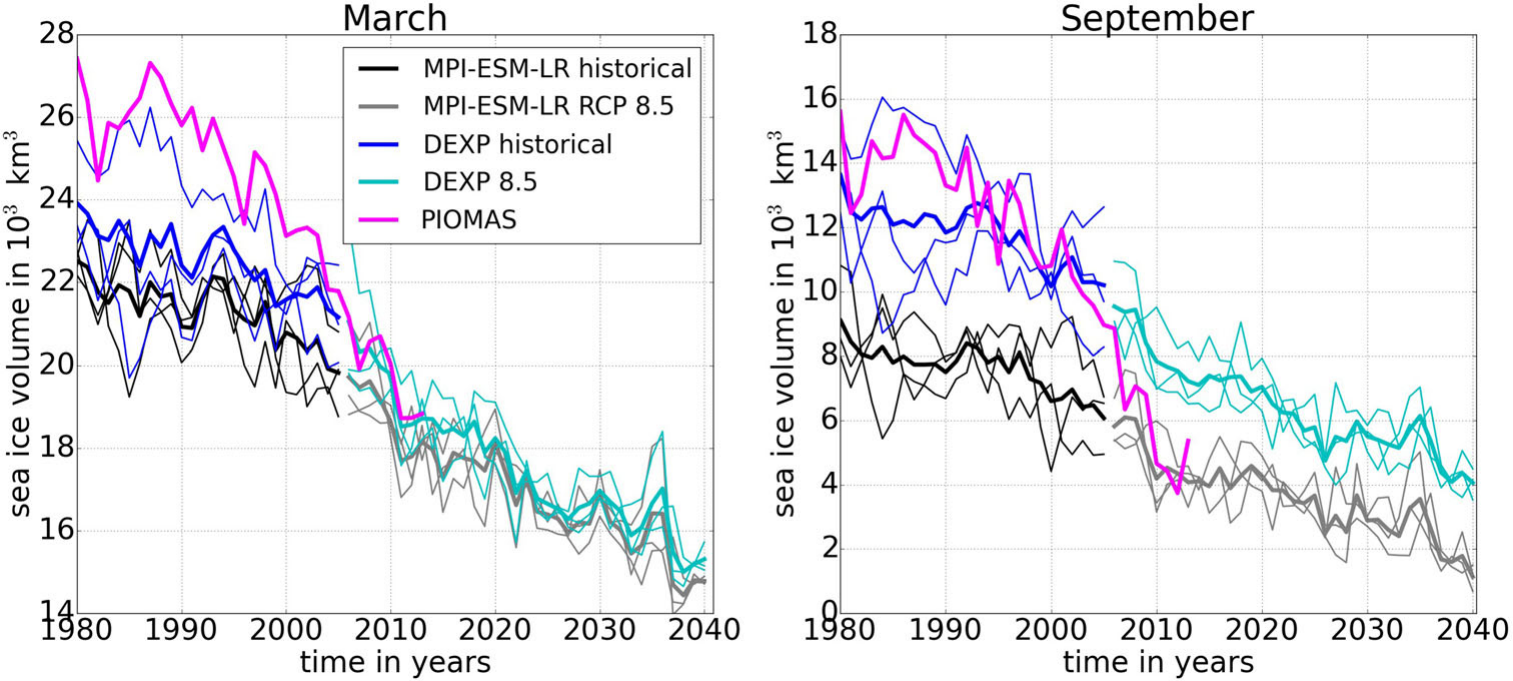
Northern hemisphere (without Bering Sea and the Sea of Okhotsk, see Electronic supplementary material for explanation) integrated sea ice volume from the MPI-ESM-LR and the downscaling experiments (DEXP8.5) for the time period 1980–2040. The RCP 8.5 simulations start in 2006. Thick lines indicate the ensemble mean. Thin lines show the individual ensemble member. PIOMAS sea ice volume is shown in magenta for comparison

So how does the development of Arctic sea ice differ between the different model experiments: the regional MITgcm, which is used for the downscaling experiment DEXP8.5, and the MPI-ESM-LR, which had been picked as the best performing of the CMIP5 models in terms of sea ice extent? In addition to the spatial resolution, the Earth system model MPI-ESM-LR and the regional downscaling model used for the experiment DEXP8.5 differ in a number of parameterizations of physical processes, which also affects the simulation of sea ice concentration, extent, and thickness. Here we find that regional downscaling model in DEXP8.5 produces thicker sea ice than the MPI-ESM-LR, leading to an overall higher sea ice volume. Integrated sea ice volume is shown in Fig. 1 for both MPI-ESM-LR and DEXP8.5. Since observations of sea ice volume (or thickness for that matter) do not exist over a longer period, both simulations are compared to results from PIOMAS version 2.1 (Zhang and Rothrock 2003). PIOMAS is a coupled sea ice–ocean model that is based on an assimilation of satellite-derived ice concentration data and sea surface temperature (Schweiger et al. 2011).^3^ PIOMAS covers the period of 1979–2016. It should be stressed that due to the natural variability of the climate system, fully coupled Earth system models cannot be expected to simulate the exact timing of observed sea ice volume fluctuations. This is different for coupled sea ice–ocean models like PIOMAS, which are driven by observed atmospheric data. The ensemble mean of DEXP8.5 (3 members) exhibits about 2000 (5000) km^3^ higher sea ice volume than the ensemble mean of MPI-ESM-LR (3 members) in March (September) (Fig. 1). Ensemble members of both sets of models exhibit a steady long-term decrease of sea ice volume in both winter and summer seasons during the historical period, and stronger fluctuations occur for shorter periods. However, the rate of sea ice volume decrease is much greater for PIOMAS than for both MPI-ESM-LR and DEXP8.5 model experiments. Both MPI-ESM-LR and DEXP8.5 model experiments show a lower winter sea ice volume than PIOMAS during the historical period. The much weaker trend for the 1990s to the 2000s might be an indication for internal variability superimposed on the greenhouse gas-induced long-term trend, but could also be a consequence of model errors.

**Fig. 2:**
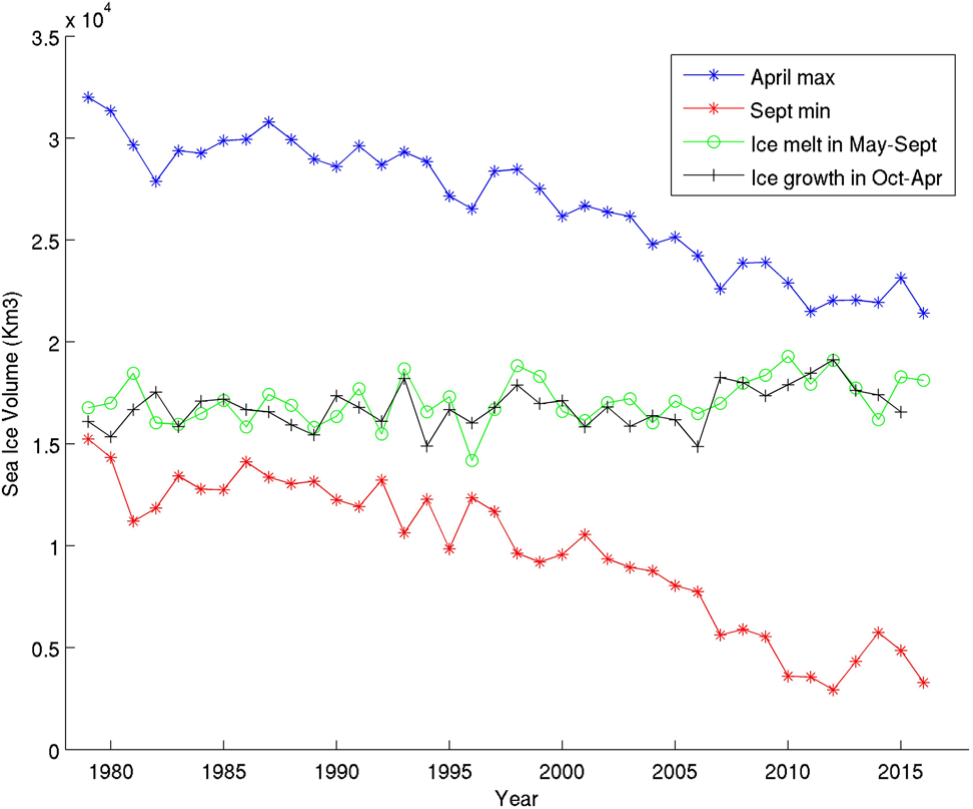
PIOMAS-simulated April (winter/blue) and September (summer/red) Arctic sea ice volume for the past 35 years. The green curve indicates the amount of sea ice melting between April and September each year. The black curve indicates the amount of sea ice freezing between September and April the following year. Results cover the entire northern hemisphere (northern hemisphere defined as encompassing Arctic and Subarctic sea ice-covered regions)

PIOMAS simulates the northern hemisphere sea ice volume seasonal cycle showing a similar trend in winter (April) and summer (September) over the past 35 years (Fig. 2). Over the past 35 years, the Arctic sea ice volume simulated by PIOMAS for April reduced from 32 000 km^3^ to about 22 000 km^3^ corresponding to a sea ice loss of 10 000 km^3^ at the end of recent winters compared to earlier winters. Remarkably, PIOMAS indicated a similar trend in summer with 16 000 km^3^ Arctic sea ice volume at the end of the melting season (September) 35 years ago, versus 4000 km^3^ during more recent years corresponding to a sea ice volume decrease of 12 000 km^3^ at the end of recent summers compared to earlier summers. According to this simulation, the total sea ice volume at the end of the summer season today is only 25% of what it was 35 years ago and corresponds to a summer sea ice extent half the size as before, combined with an overall thinning of sea ice by half over the same period.^4^

From the winter sea ice volume maximum and the summer sea ice volume minimum, we can easily deduce the ice growth from October to April and the ice melt from May to September, for each year over the past 35 years. It is interesting to compare sea ice growth (black curve in Fig. 2) from sea ice melt (green curve in Fig. 2). Both sea ice growth and sea ice melt are slightly increasing by about 2000 km^3^ over the 35-year period indicative of a stronger seasonal cycle. Still, compared to the sea ice loss both at the end of the winter (10 000 km^3^) and at the end of the summer (12 000 km^3^) over the same period of time (past 35 years), this increase for sea ice growth and sea ice melt is 5–6 times less and is simply resulting from the fact that there is more and more open water to freeze up in each fall–winter season.

**Fig. 3:**
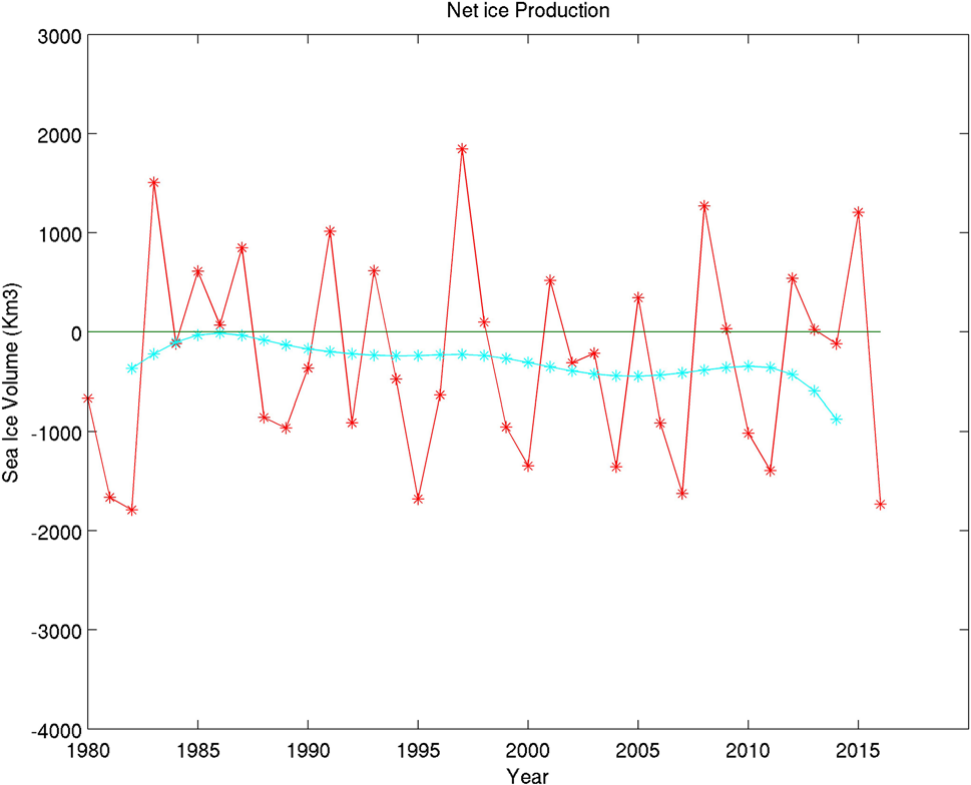
PIOMAS net sea ice production over the past 35 years computed from sea ice growth minus sea ice melt every year (i.e., the black curve minus the green curve in Fig. 2). The cyan curve represents the 5-year running mean values. The red curve really demonstrates the importance of the sea ice volume interannual variability superimposed over the long-term trend (cyan curve). Results cover the entire northern hemisphere (northern hemisphere defined as encompassing Arctic and Subarctic sea ice-covered regions)

Another interesting result is shown in Fig. 3 indicating the net sea ice production obtained by subtracting ice melt from ice growth each year (i.e., subtracting the green from the black curve in Fig. 2). The 5-year running mean values (green curve in Fig. 3) indicate a negative net sea ice mass balance starting from the mid-80s and slightly enhancing up to the present with a rate of a few 100 km^3^ every year over the past 35 years superimposed to a 4-year oscillation. This results from the fact that the sea ice growth is increasing at a lower rate than the sea ice melt, creating a net negative sea ice mass balance. For most of the 35-year period, the net ice production is negative since the mid-80s when it was at equilibrium. The main reason for the drastic sea ice loss cannot be attributed to a moderate melt increase in summer but rather to a drastic reduction in winter sea ice growth from October to April each year and that cannot compensate for sea ice melt from May to September anymore.

**Fig. 4:**
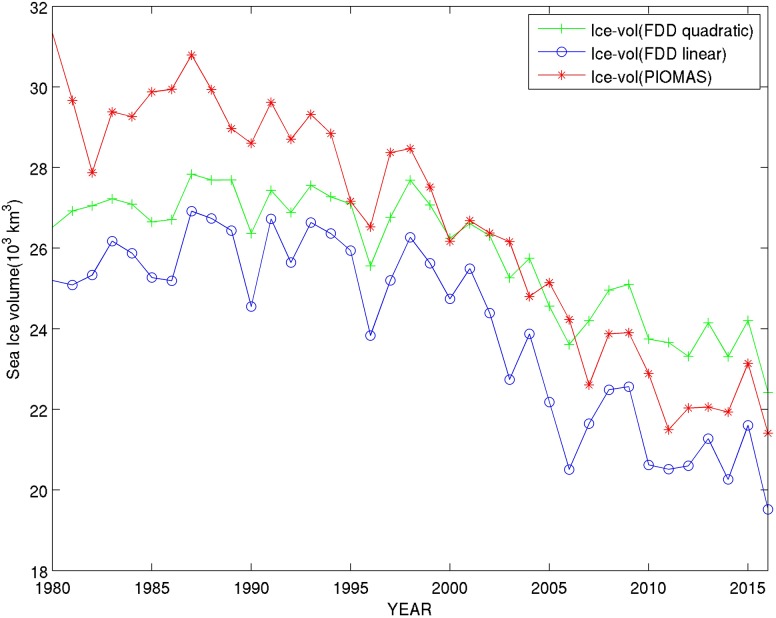
Comparison between PIOMAS sea ice volume in April each year over the past 35 years and sea ice volume based on Freezing Degree Days (FDD) using either a linear or a quadratic approximation from which sea ice volume production can be estimated. Sea ice volume is calculated using the spatial distribution of FDD based on ERA-Interim 2-m air temperature reanalysis data covering the whole northern hemisphere

Since PIOMAS is assimilating surface air temperature (Schweiger et al. 2011), it is interesting and useful to calculate the amount of sea ice volume directly resulting from freezing during the entire freezing period extending from September to October until April to May the following year, for each year. Based on the 2-m standard air temperature provided by the ERA-Interim reanalysis, we calculated the number of Freezing Degree Days (FDD) over the entire Arctic Ocean for the past 35 years.^5^ During the early 80s, the amount of sea ice calculated from FDD was significantly lower than the sea ice volume produced by PIOMAS in winter (Fig. 4). This was due to the abundance of multiyear ice (MYI) during the early 80s that FDD could not account for. During more recent years, PIOMAS and FDD estimations are both producing a similar amount of sea ice. This is a remarkable result. The similarity is due to a disappearance of MYI during recent years as also confirmed by the better fit for the FFD linear approximation with PIOMAS rather than the quadratic approximation, which is more appropriate for thicker ice. Remaining differences between the two could be attributed to the ocean heat flux not taken into account in sea ice volume based on FDD, but also likely due to model errors. The ocean effect is particularly important in the marginal ice zone.

The similarity between PIOMAS and FDD sea ice volume winter production, including the long-term trend as well as the interannual and interdecadal variability, is striking. This not only shows the robustness of the used methods, but also suggests that in the northern hemisphere as a whole, the surface air temperature driving freezing is a major factor for the sea ice volume development, as reflected in both the PIOMAS and the FDD-based sea ice volume estimations. The reason that PIOMAS and FDD winter ice volumes match each other after 2000 is likely the fact that the ice cover is very thin throughout the year in much of the Arctic Ocean. The albedo feedback in summer plays a significant role in ice melt and in storing heat in the upper ocean. When fall comes, the heat stored in the upper ocean gets lost through leads and tends to suppress ice growth. Thinner ice responds to cooling temperature in a more linear fashion because heat conduction in thin ice is more of a linear process. This means that winter ice growth is more closely correlated with air temperature. Thus, the closeness of PIOMAS sea ice volume and FDD-based sea ice volume in variability and magnitude in recent years indicates that the air temperature change has been the dominant factor at the pan-Arctic scale.

If the trend for the past 35 years continues, then both PIOMAS and FDD suggest that a complete Arctic sea ice melting would more likely occur within a 10-year time frame compared to a 20-year time frame for MPI-ESM-LR and DEXP8.5, both exhibiting a much weaker trend in the recent past one or two decades. Both PIOMAS and FDD use 2-m air temperature, which has an increasing trend likely linked to natural variability and anthropogenic forcing. The change in ice volume (winter growth versus summer melt) corresponds to Arctic warming superimposed to natural interannual variability. There is more likely no precise time prediction when Arctic will be summer ice free because of the interannual fluctuations. The natural variability of the Arctic sea ice cover, superimposed on a long-term trend due to anthropogenic climate change, plus model uncertainties, makes a prediction of a precise timing for a quasi-ice-free Arctic in summer uncertain. This kind of prediction is marginally important compared to the real situation where 75% of the Arctic sea ice has already melted away at the end of the summer. More likely, it will not take very long (one or two decades) to melt away the remaining 25% of Arctic sea ice by the end of the summer season and this is what really matters for a number of critical issues such as shipping activities and other human activities across the whole Arctic Ocean.

## Future sea ice evolution along the Northeast passage

**Fig. 5:**
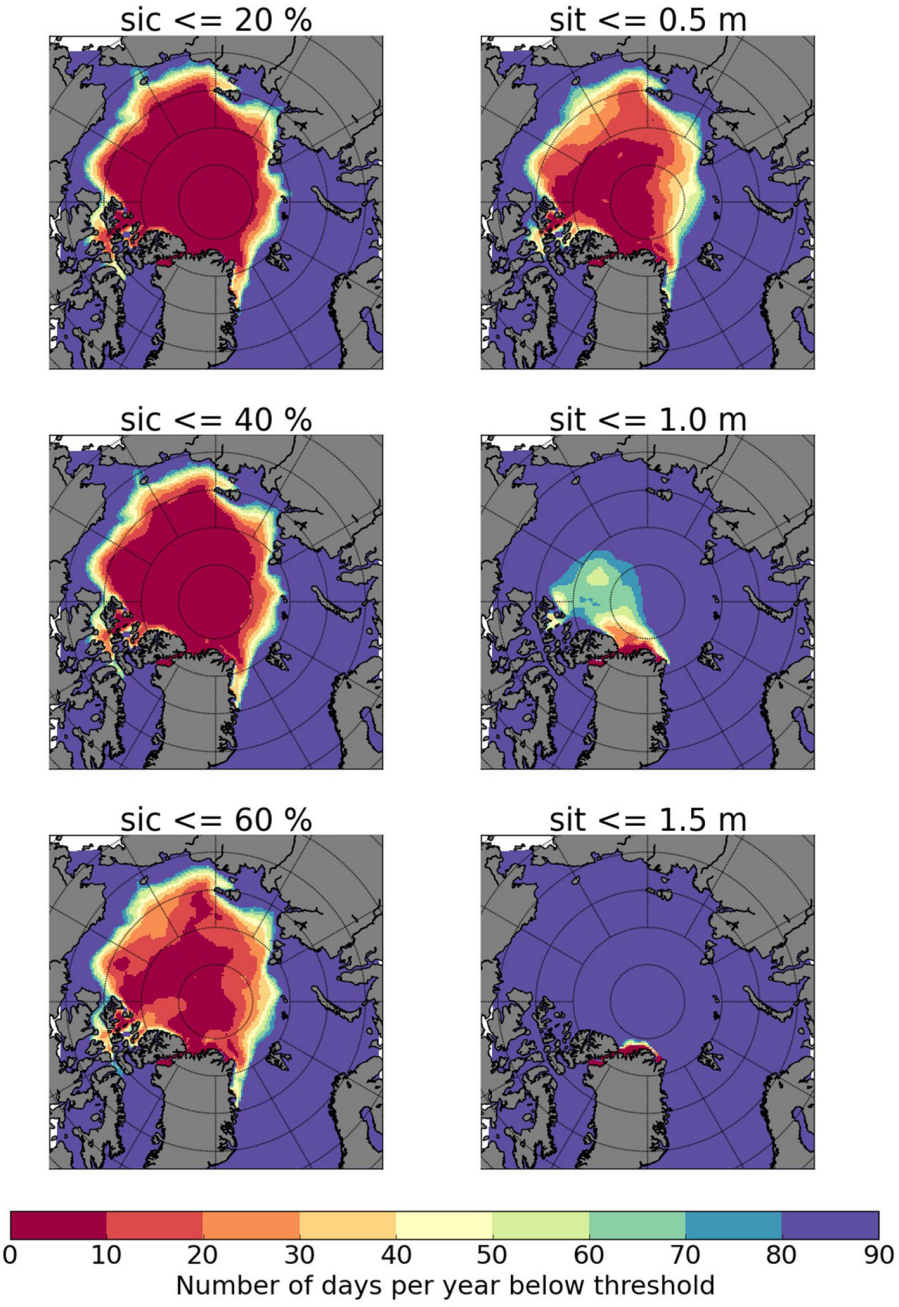
DEXP8.5 ensemble mean of the number of “low ice condition” expected days per year for the mean over 2026–2040. “Low ice conditions” are defined as daily mean sea ice concentration (sic) < 20, 40 and 60% and daily mean sea ice thickness (sit) < 0.5, 1.0 and 1.5 m

In addition to helping improve the understanding and prediction of the coupled climate system, model simulations in the framework of ACCESS have been used to provide guidance on what range of future sea ice conditions may be encountered in areas where it affects human activities, such as shipping. Here we make use of the DEXP8.5 experiment with finer spatial resolution, zooming into the regions most relevant for long-distance shipping in the Arctic. We focus on the sea ice conditions in near-coastal areas and along potential shipping routes, in terms of sea ice concentration, thickness, and the length of periods with ice conditions that are favorable for shipping. As different ship types have different limits regarding their capability to navigate in different sea ice thickness and concentration conditions, we analyze the number of days for which sea ice thickness (sit) and sea ice concentration (sic) fall below specific thresholds relevant for shipping. The analysis is based on daily mean sic and sit from experiment DEXP8.5. Figure 5 shows the number of days per year below these thresholds as an ensemble mean over the period 2026–2040. The model experiment suggests that there will be a considerable lengthening of the navigable season. For example, along the NEP in the period 2015–2040 there are more than 40 days with sic below 40% and sit below 0.5 m^6^ (Fig. 5). However, the number of days is the sum of all ‘low ice condition’ days, which are not necessarily taking place in a row, so to allow easy passage.

**Fig. 6:**
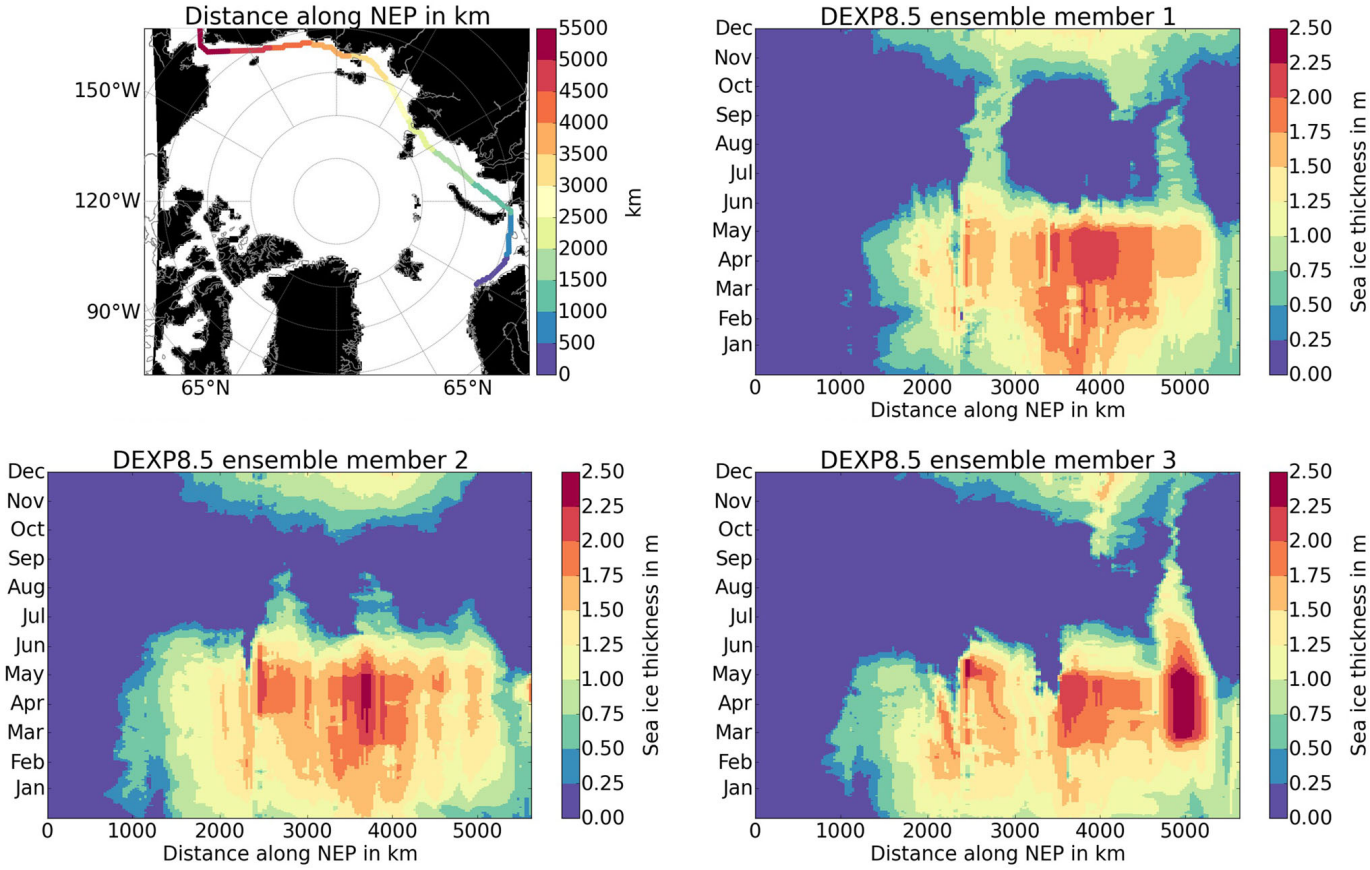
Sea ice conditions along the NEP. The upper left panel illustrates the path of the NEP with the distance traveled from Northern Norway to the Bering Strait in color code. The remaining three panels illustrate model projections of the daily sea ice thickness in 2040 for each of the three ensemble members of DEXP8.5 along this path. The vertical axis shows time (month), and the horizontal axis shows the distance along the NEP from the starting point in Northern Norway (left) to the Bering Strait (right)

**Fig. 7:**
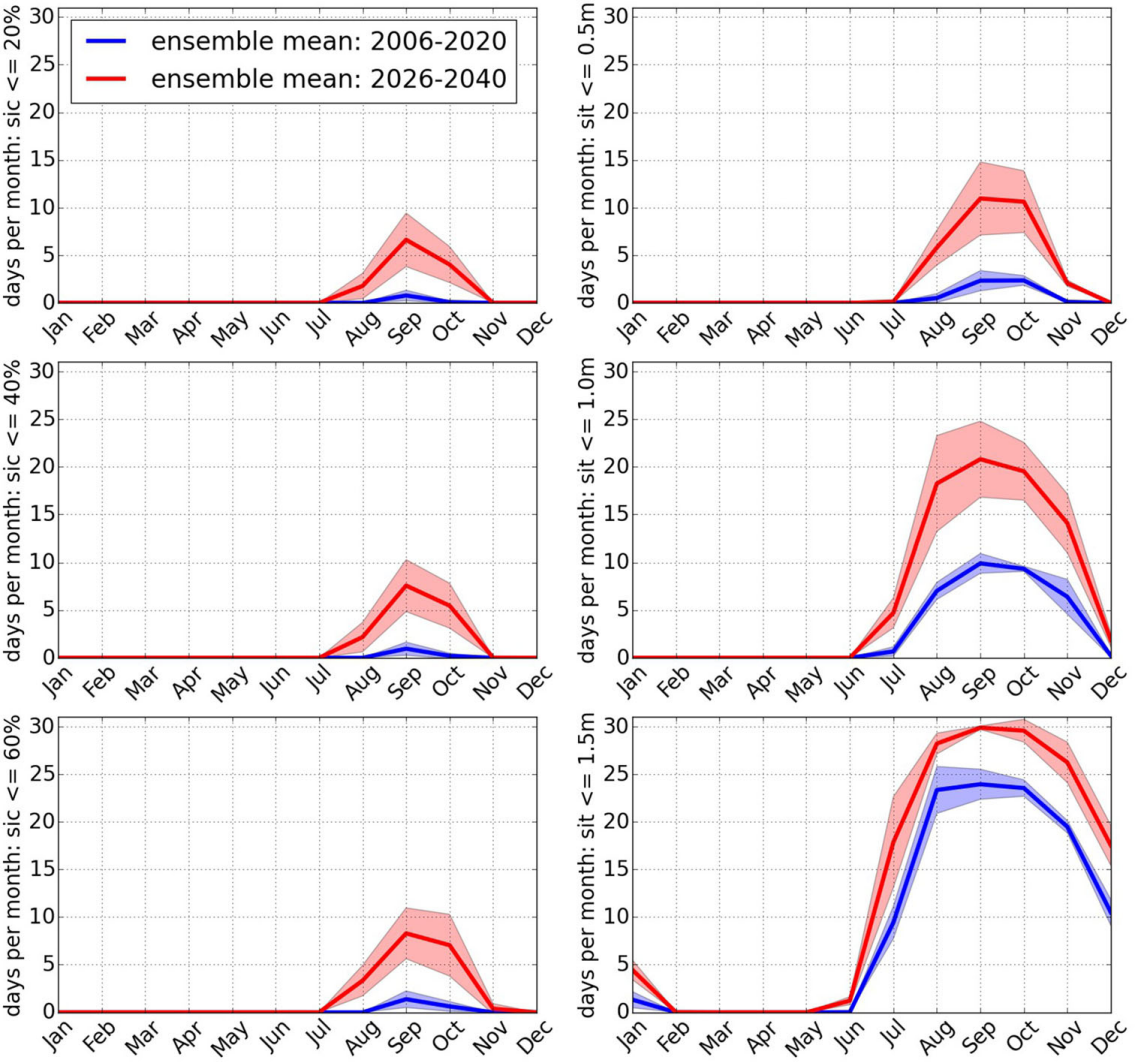
Number of days per month below threshold. A day is counted if the sea ice variable is below its threshold along the entire Northeast Passage. The thick line denotes the ensemble mean. The shaded area indicates the ensemble spread and is a measure for the internal variability in the system

An alternative view on the ice conditions as they are relevant for shipping is presented in Fig. 6. It shows the daily sea ice thickness of DEXP8.5 for each ensemble member along the NEP for the year 2040. The graphs exemplify that despite the largely ice-free passage in each of the three ensemble runs over an extended period of one to three months in summer, there may be still blockages at key locations. Those are the Vilkitsky (km 2600) and the Dmitry–Laptev Strait (km 5000), in or east of which even thin and mobile ice may pile up or be stacked to block the passage. While it is unclear how likely such blockages will be in a future Arctic, Gerdes and Köberle (2007) speculated that persistent large-scale wind patterns in the Earth system models may lead to such situations, too, and it is unclear how realistic those might be. The results presented in Fig. 6 also show the large internal variability in the system, leading to different realizations of the sea ice situation despite the same forcing for the three ensemble members. Figure 7 shows a different way to understand the sea ice development along the NEP, by presenting the number of days with sic and sit being below a certain threshold along the entire NEP contrasting the period 2006–2020 with 2026–2040. The mean number of days with «low ice conditions» as well as the ensemble spread increases considerably for the later time period. The «low ice» period starts one month earlier and ends one month later in the later time period. Moreover, the length of the period with sit < 1.5 m increases by four months, lasting from May to February.

The model results and the analysis in terms of parameters relevant for shipping suggest that despite an overall increase of navigability in the Arctic, still a lot of variability of sea ice cover needs to be taken into account over the coming decades. This will make a reliable weather forecast, which is also the prerequisite for a short-term sea ice prediction, even more important.

## Improving weather forecasting for operational activities in the Arctic

The possibilities in the Arctic for increasing activities such as ship traffic and resource exploitation also mean that operators need to deal with risks related to the physical Arctic environment (weather, ocean, sea ice). Operational challenges include risk factors such as low temperature, occurrence of high winds, fog, and darkness during the winter season. Marine operations might additionally be influenced by ocean waves, icing from sea sprays, and the presence of sea ice and icebergs. These environmental factors may occur in combination, thus increasing the operational challenges. In remote areas, the infrastructure and capability to manage difficult situations, hazards, or accidents may be distant or even unavailable in many cases.

This emphasizes a need for risk management for operators in the Arctic (Emmerson and Lahn 2012). Weather forecasting is one element of risk mitigation, helping operators to have knowledge of weather-related risk in advance through forecasting capabilities at time ranges from a few hours to days ahead. Weather forecasts are also input to ice forecasting, so it contributes to dealing with risks connected to ocean conditions.

Forecasts from short-range Numerical Weather Prediction (NWP) models are the main tool in such forecasting. Work undertaken at the Norwegian Meteorological Institute (MET Norway) as a part of ACCESS has assessed the forecasting capabilities in the Atlantic sector of the Arctic by investigating the forecast performance of the global ECMWF (European Centre for Medium Range Weather Forecasting) NWP and the regional HIRLAM (High Resolution Limited Area Model) model previously run at MET Norway. Pressure at mean sea level (MSLP) is a meteorological quantity not easily felt, unlike wind or temperature, but still the pressure field is closely connected to the weather systems. For instance, away from topography, the wind is closely connected to the pressure gradient through the so-called geostrophic relation. Pressure is much less influenced by local topographic and surface conditions than wind or temperature, and thus comparing pressure verification statistics at different locations is very well suited for getting a picture of how the NWP models are able to capture the main large-scale weather system variations.

**Fig. 8:**
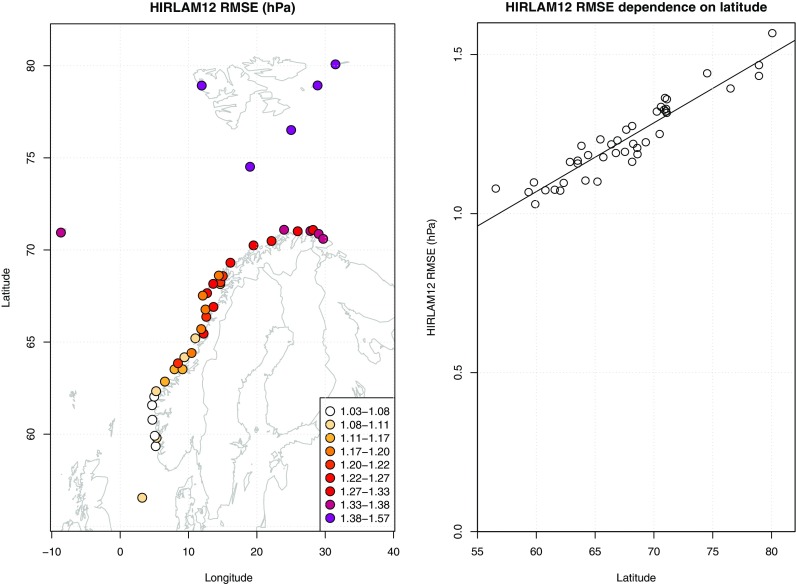
Decreasing quality of weather prognosis going northward illustrated by the root mean square errors (hPa). Left: Errors in pressure (root mean square errors vs observations) for HIRLAM forecasts in the range from 18 to 42 h. Right: The same dataset with root mean square error in pressure (hPa) for forecasts against latitude

Figure 8 shows root mean square errors for MSLP of the HIRLAM regional NWP model. For this sector of the Arctic, there is a striking general decrease in forecast quality when moving northwards. This is seen in spite of the fact that the day-to-day pressure variability (not shown here) is comparatively small at the highest latitude stations.

A candidate for explaining the decline in the quality toward the North would be the corresponding decrease in the observation density of the conventional meteorological observing network. The NWP forecasts depend on determining the initial state of the atmosphere using observations in the so-called data assimilation. For surface observation data, there is a general gap in pressure observations over parts of the sea ice domain and parts of the ocean domain as there is only limited coverage from drifting buoys. There is almost no coverage of near-surface wind observations over sea ice. The wind coverage over ocean is good due to satellite scatterometers providing surface wind observations (see, e.g., Figa-Saldana et al. 2002) and over populated continents due to conventional surface meteorological stations. Surface pressure gradients and near-surface winds are closely linked in the Arctic through the geostrophic relationship. However, where only pressure gradient information is available through wind observations, the absolute value of the pressure field would need to be settled thanks to an extended coverage of surface pressure information.

During the period of the ACCESS project, a novel state-of-the-art convection resolving regional NWP system covering a high-latitude area, the so-called AROME-Arctic model, was implemented at MET Norway. This is a version of the HARMONIE-AROME^7^ model and runs at a horizontal resolution of 2.5 km grid length. For applying observations to update the model initial state for the forecasts, the AROME-Arctic model uses three-dimensional variational assimilation system (3D-VAR) for atmospheric and optimum interpolation (OI) for surface data assimilation. This system was used to perform observing system experiments (OSE) including all available surface and upper-air conventional observations and radiance observations from NOAA and METOP satellites. These experiments indicated that

1. the present upper-air conventional (radiosondes, aircraft) observations in the area are too scarce to have a significant effect on forecasts;
2. satellite remote sensing data for vertical profiling of the atmosphere (sounding data) play an important role in improving forecasts quality. This clearly highlights that satellite information will be important also in the future evolution of the Arctic observing system and that enhancing the extraction of information from such satellites could be a key area.

All the satellite sensors mentioned above are on polar-orbiting satellites, which have the property that the density of the ground tracks increases poleward, thus giving good coverage in the Arctic. Our ability to benefit from satellite information in analyses and forecasts of numerical weather prediction systems evolves with time, and there is a potential for further enhancing our methods for exploiting already existing satellite sensors. Efforts in this direction would not require new satellite programs and could be a way of improving forecasting with further research at relatively limited cost. Key areas which we believe have a potential for improving information from satellite remote sensing (sounding) instruments for NWP in the Arctic are as follows:

1. Accounting for the surface contribution to the signal over sea ice: Sounding channels with significant contribution to the measured signal from Arctic Ocean sea ice surfaces are usually rejected in the data assimilation. If we could account for the contribution from the surface by drawing information on emissivity and emitting temperature in new ways, it will enable us using more channels providing temperature and moisture information in the lower troposphere (see, e.g., Tonboe et al. 2013; Karbou et al. 2014).
2. Better ability to describe and account for the cloud contribution to the signals: Clouds are ubiquitous in the Arctic and assimilation of satellite sounding data relies on a « cloud clearing » procedure before assimilation which has a goal to only allow assimilation of channels without cloud contribution to the signal. There is a potential for improving the reliability of cloud clearing and also for novel methods to exploit the cloudy signals (following, for instance, the ideas for «all-sky» assimilation demonstrated by Geer et al. 2010).

Finding scenarios for new satellite missions or programs is expensive and subject to well-established long-term processes and thorough investigations in the international satellite agencies. One can hope that further evolution of the satellite observing system will help fill the observation gap in the Arctic, but this can only happen in the long term (WIGOS 2013). Here we will mention some possible extensions of the conventional observing network, which could be practically and even economically realistic to implement in a shorter time range, although it would still require some funding scheme and possibly international cooperation.

When looking for options for such extensions, it is attractive to look for alternatives with the lowest cost relative to the impact on forecast. There is extensive literature on the impact of observations or observation types on NWP forecast quality; however, few studies consider also the cost of each observation to rank observation types according to impact per cost. Nevertheless, Eyre and Reid (2014) made the first attempt to investigate this in a global forecasting context. This study pointed to many uncertainties and needs for refinements of the methodology, but the results showed drifting ocean surface buoys to be the conventional observation type with the largest impact per cost globally (see also Andersson and Sato 2012). This is probably related to the fact that they are often deployed at locations where few other observations are available. Also conventional aircraft observations showed good impact per cost, and radiosondes showed moderate impact per cost on average.

The three-dimensional nature of the atmosphere and previous experience certainly indicate that profile information (sounding) is important. A relatively cheap way to obtain more conventional sounding data is to perform more frequent radiosonde launches at already existing launch sites (at least twice per day or four times per day). There are a few existing radiosondes stations with staff and necessary infrastructure at some remote Arctic islands. Since basic infrastructure for these launch sites are already in place, increasing the number of launches per day at these sites could be a good solution to provide cost-efficient extended data coverage. Our investigation showed that guaranteeing at least two launches per day of radiosonde at already existing sites provides benefit for the updated initial state (analysis) and forecasts of geopotential and humidity, while four times launch per day provides a further significant beneficial impact on analysis and forecast of all verified parameters.

Based on the indications from Eyre and Reid (2014), we should look at the option of increasing the number of surface buoys. Even if that study was for observations globally, it seems reasonable to also expect a good impact of these observations in the Arctic, based on the decrease in the observation density of the conventional meteorological observing network when going northwards, and lack of satellite information on surface pressure. Deploying extra buoys in the Arctic has been done in campaigns in the past, so it should be possible to implement it if more funding is made available through some permanent program. Scenarios could include increasing the number of drifting surface buoys measuring the air pressure in the Arctic domain by factors two, three, or four. Surface pressure information is important to assimilation for the dynamics of the atmosphere and such buoys are usually also equipped with temperature sensors providing additional information of interest. We tried to define the optimal network distribution based on the accuracy of the forecasts issued from the analyses using the different scenarios for surface buoy networks. Increasing the number of buoys by a factor of four did not show any overall improvements in accuracy of forecasts for mid- and high troposphere compared to forecasts issued from analyses using three times more buoys. Meanwhile, the accuracy of forecasts issued from the analyses using two times more surface buoys is less than that of those issued using three times more buoys. We conclude that three times more buoys represents a cost-efficient distribution of buoys inside our model domain in the context of our state-of-the-art regional forecasting system.

## Conclusion

As far as Arctic navigation is concerned, sea ice extent, sea ice concentration, and sea ice thickness really matter. From a climatic point of view, sea ice volume combining sea ice extent, concentration, and thickness is the key element. Based on one of the most pertinent IPCC Earth system models for predicting Arctic sea ice extent, concentration, and thickness and referring to the best available Pan-Arctic Ice-Ocean Modeling and Assimilation System (PIOMAS) and newly estimated sea ice volume deduced from Freezing Degree Days (FDD), we evaluated potential model predictive capacity for supporting marine transportation across the Arctic Ocean for the next three decades. Both PIOMAS and FDD confirm a drastic Arctic sea ice volume loss of about 75% at the end of the summer season (September) when comparing sea ice volume for recent years with those obtained 35 years ago. This results from a 50% reduction of both sea ice extent and sea ice thickness over the entire Arctic Ocean and over a 35-year time period. Both PIOMAS and FDD sea ice volume-based estimations confirm that the drastic Arctic sea ice volume reduction (and consequently sea ice extent and sea ice thickness) is mainly due to a decrease in winter ice growth which does not compensate for ice loss in summer ice melt anymore. CMIP5 climate simulations confirm the trend for sea ice reduction during most of the years extending over the next 30 years facilitating marine transportation across the Arctic Ocean. Earth system models still have deficits though. The long-term trend of sea ice volume indicated in the assimilated PIOMAS model is stronger than that found in the Earth system models used in the current project. It is unclear how much of the PIOMAS trend is due to climate change and how much is due to internal variability which Earth system models would not necessarily show in the same time period. Observational data show that sea ice variability at the interannual and intradecadal time scale is quite significant. The same variability is not yet properly simulated by Earth system models. This even holds for the seasonal cycle. Earth system models need to improve sea ice production during winter and sea ice melting during summer, also impacting sea ice thickness. Finally, higher resolution is clearly needed when it comes to support planning safe marine transportation across the Arctic Ocean along the Northeast Passage (NEP) and/or the Northwest Passage (NWP), e.g., when taking into account critical narrow passages for ship navigation. Weather forecasts for operational use are also suffering from undersampled radiosondes coverage that is falling drastically at high latitudes. For supporting Arctic operations with better weather forecasting capabilities in the near future, it is recommended to improve both regional and seasonal coverage of drifting buoys by a factor of 3 and an increase of operation/launch of radiosondes to 4 times per day.

## Electronic supplementary material

Below is the link to the electronic supplementary material.

Supplementary material 1 (PDF 1009 kb)

## Footnotes

1 The CMIP5 models are coupled Earth system models covering long time periods for a large number of experiments and ensemble simulations. Earth system model simulations were performed in ensembles, the members of which differ only by a small perturbation at the beginning of the experiment, to cover the range of natural variability. The external forcing, such as greenhouse gas concentrations, was the same for each ensemble member, and long-term trends due to these, for example, should be detectable in the model experiments. CMIP5 models thus provide the ‘big picture.’

2 The regional Arctic model is based on the MITgcm (Massachusetts Institute of Technology general circulation model; Marshall et al. 1997). (For technical details regarding our Arctic version of the MITgcm, see Castro-Morales et al. (2014)). The experiments for the future (DEXP8.5: 2006–2040) we present here are based on the «representative concentration pathway» (RCP) emission scenario RCP 8.5 (Moss et al. 2010).

3 Data assimilation is a method to modify a model simulation such that it comes as close as possible to a set of observed data. Since the model produces a physically consistent set of data, the constraints put on the model system by the observed variables is deemed to improve also the variables which are not assimilated from an observational counterpart.

4 Sea ice export out of the investigated domain does not contribute to these calculations since the domain is large enough to encompass all areas of sea ice formation and melt.

5 In order to calculate sea ice volume based on freezing degree days, we used the same approach as Harpaintner et al (2001). Two approximations (linear and quadratic) were tested. The linear approximation is more adapted to thinner ice, while the quadratic approximation is more appropriate for thicker ice. It needs to be noted that volume estimations based on FDD are only able to calculate the newly formed ice or First Year Ice (FYI) and cannot take into account any multiyear ice (MYI) and/or deformed ice.

6 The thresholds for sic and sit which are to be found along the NEP are important data for the planning of shipping in sea ice-covered waters (pers. comm. Nils Reimer, Hamburgische Schiffbau-Versuchsanstalt (HSVA)).

7 HIRLAM–ALADIN Research on Mesoscale Operational NWP in Euromed, see Bengtsson et al. 2017.
