# Supplementary material for: Future sea ice conditions and weather forecasts in the Arctic: Implications for Arctic shipping
Source: Ambio. 2017 Oct 27;46(Suppl 3):355–67. doi: 10.1007/s13280-017-0951-5 (PMC5673867; doi:10.1007/s13280-017-0951-5)
Supplement: Supplementary file 1 — Supplementary material 1 (PDF 1009 kb) [file 13280_2017_951_MOESM1_ESM.pdf]

Ambio

Supplementary Material:

Title: Future sea ice conditions and weather forecasts in the Arctic: Implications for Arctic shipping

Authors: Jean-Claude Gascard, Kathrin Riemann-Campe, Rüdiger Gerdes, Harald Schyberg, Roger Randriamampianina, Michael Karcher, Jinlun Zhang, Mehrad Rafizadeh

Method on selecting CMIP5 models

34 CMIP5 models have been analyzed during the ACCESS project (Riemann-Campe et al., 2014). For the Arctic regions these models provide a large range of possible future environments, leading to the question which of these simulations can be deemed as the most realistic. One way to attempt at an answer is to sub-sample these model experiments to make use just of those, which perform best in comparison with observed historical data. Overland et al. [2011] show that sub-sampling CMIP models, by comparing selected variables to observational data in specific regions reduces the root mean square error of these variables considerably. Since sea ice concentration derived from satellites provides the longest, large-scale 'observational' record, it has been used for sub-sampling CMIP3 and CMIP5 models in previous studies (e.g. Massonnet et al. (2012) and Wang and Overland (2012)). We also use satellite derived sea ice concentration to select the better performing CMIP5 models with regard to northern hemispheric sea ice distribution and intra annual variability.

We compute the misfit of the simulated seasonal cycle of the sea ice area in comparison to satellite-derived data for a number of Arctic regions. This method is based on the study by Overland and Wang [2007]. Furthermore, we weigh the misfit with a function based on the varying uncertainty of Satellite measurements during the annual cycle, using two different satellite-derived data sets (by OSI SAF (Ocean and Sea Ice Satellite Application Facilities)[1] for 1979-2005 and CERSAT

---

[1] OSI SAF (Ocean and Sea Ice Satellite Application Facilities) Global sea ice concentration reprocessing dataset 1978-2009 (v1.1, 2011); Norwegian and Danish Meteorological Institutes. Available from http://osisaf.met.no.

(Centre ERS (European Remote Sensing) d'Archivage et de Traitement)[2] for 1992-2005, respectively) showing the sensitivity of the misfit due to the 'observations' [Riemann-Campe et al., 2014]. Our comparison of CMIP5 models with satellite-derived northern hemispheric sea ice concentration leads to a short list of four better performing CMIP5 models: MPI-ESM-LR, CCSM4, GFDL-CM3 and Nor-ESM1-ME (Table S1). The full comparison including the complete list of all models can be found in Rieman-Campe et al. (2014) and Petrick et al. (2017). We further chose the best performing model, MPI-ESM-LR, for our downscaling experiment DEXP.

[2] CERSAT Centre ERS (European Remote Sensing) d'Archivage et de Traitement at IFREMER, Plouzane, France) covering 1991-present. Available from ftp://ftp.ifremer.fr/ifremer/cersat/products/gridded/psi-concentration/data/arctic/

Table S1 List of CMIP5 models analysed including modeling groups and their terms of use:

Modeling Center (or Group)	Institute ID	Model Name
National Center for Atmospheric Research	NCAR	CCSM4
NOAA Geophysical Fluid Dynamics Laboratory	NOAA GFDL	GFDL-CM3
Max-Planck-Institut für Meteorologie (Max Planck Institute for Meteorology)	MPI-M	MPI-ESM-LR
Norwegian Climate Centre	NCC	NorESM1-ME

Output from yellow highlighted models is available for unrestricted use. Output from the others may only be used for non-commercial research and educational purposes. [See complete "Terms of Use": http://cmip-pcmdi.llnl.gov/cmip5/terms.html].

Additional information on model resolution

The following figures S1 to S4 show the horizontal resolution of the CMIP5 sea ice models represented by seawater depth in m. The coarse resolution along the Siberian shelf becomes apparent with narrow (less than 3 grid boxes wide) or even closed straits (figures S2 and S4) along the Northern Sea Route and the Canadian Arctic Archipelago. The simulation of flow of water and sea ice through straits is only possible if the strait has the width of minimum 3 grid boxes side by side for a

C-grid and 2 grid boxes side by side for a B-grid. The technical term B- and C- grid were defined by *Arakawa and Lamb* [1977] and relate to the position of scalar and vector variables within a model grid box. The models MPI-ESM-LR and NorESM1-ME use a C-grid and thus need 3 grid boxes side by side to enable geostrophically balanced flow through a straight. The B-grid formulation is used by the models CCSM4 and GFDL-CM3. They only need 2 grid boxes side by side to simulate geostrophically balanced flow through straights.

Figure S5 shows the horizontal resolution of the downscaling experiment DEXP8.5 for comparison. DEXP8.5 is done by a regional Arctic sea ice-ocean model. The model domain of DEXP8.5 does not cover the Bering Sea and the Sea of Okhotsk.

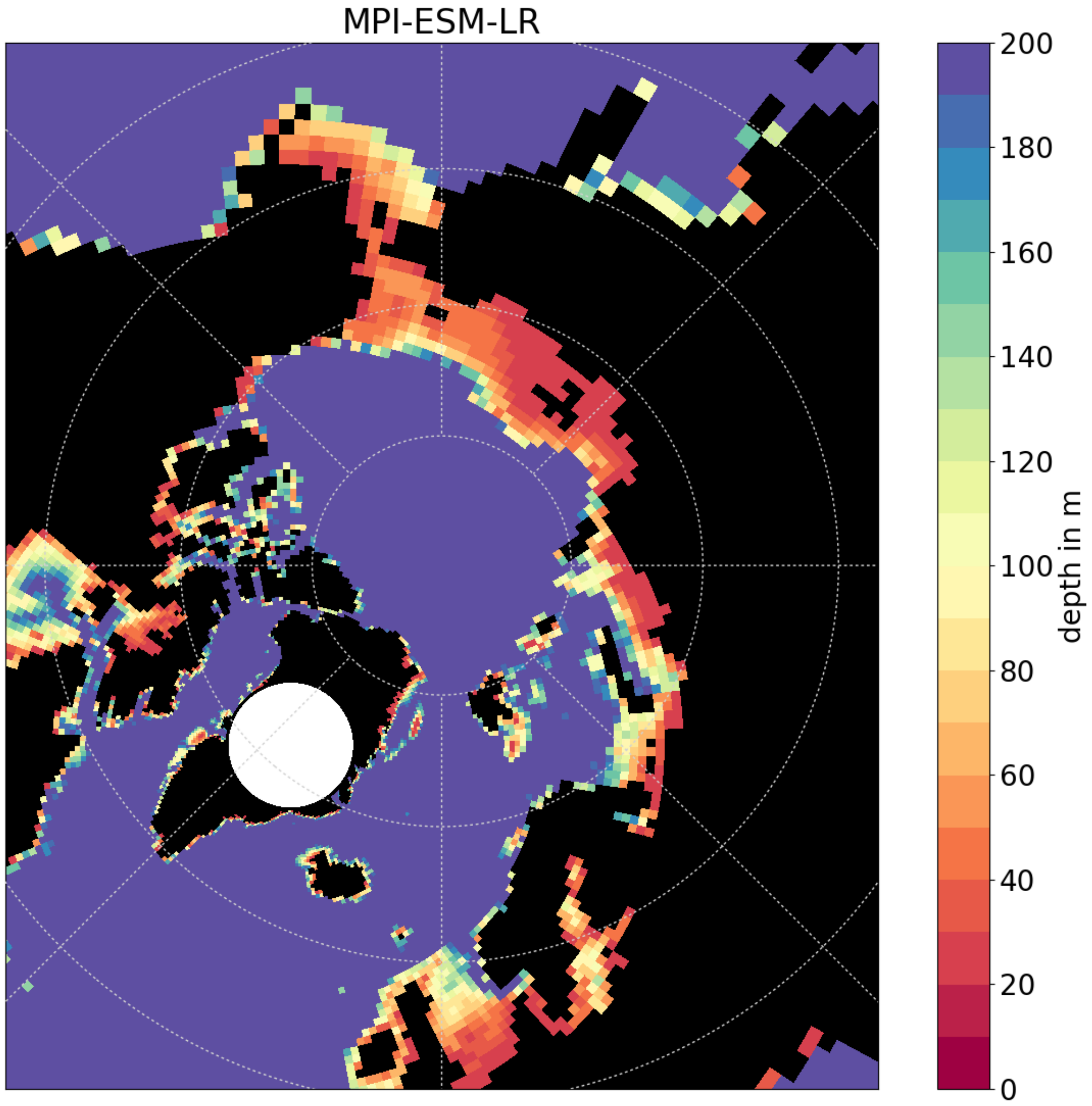

Figure-S1: Ocean depth in m of the CMIP5 model MPI-ESM-LR. Black denotes land, white indicates undefined area in model grid. The ocean and the dynamical part of the sea ice model in the MPI-ESM-LR has a horizontal resolution of 265x220 grid boxes and uses a C-grid [Jungclaus et al., 2013].

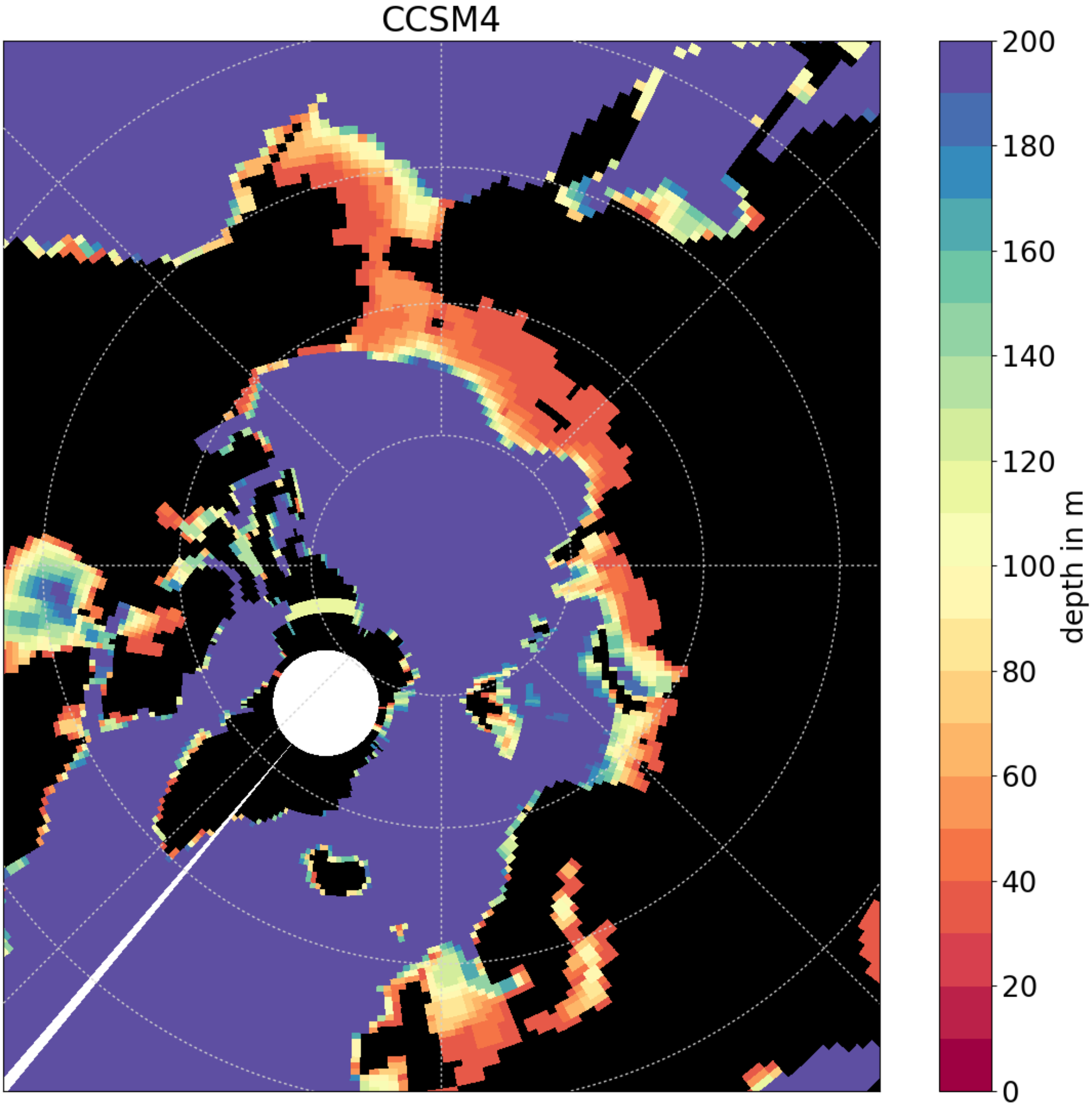

Figure-S2: Ocean depth in m of the CMIP5 model CCSM4. Black denotes land, white indicates undefined area in model grid. The ocean and sea ice model in the CCSM4 has a horizontal resolution of 320x384 grid boxes and uses a B-grid [Hunke et al., 2015].

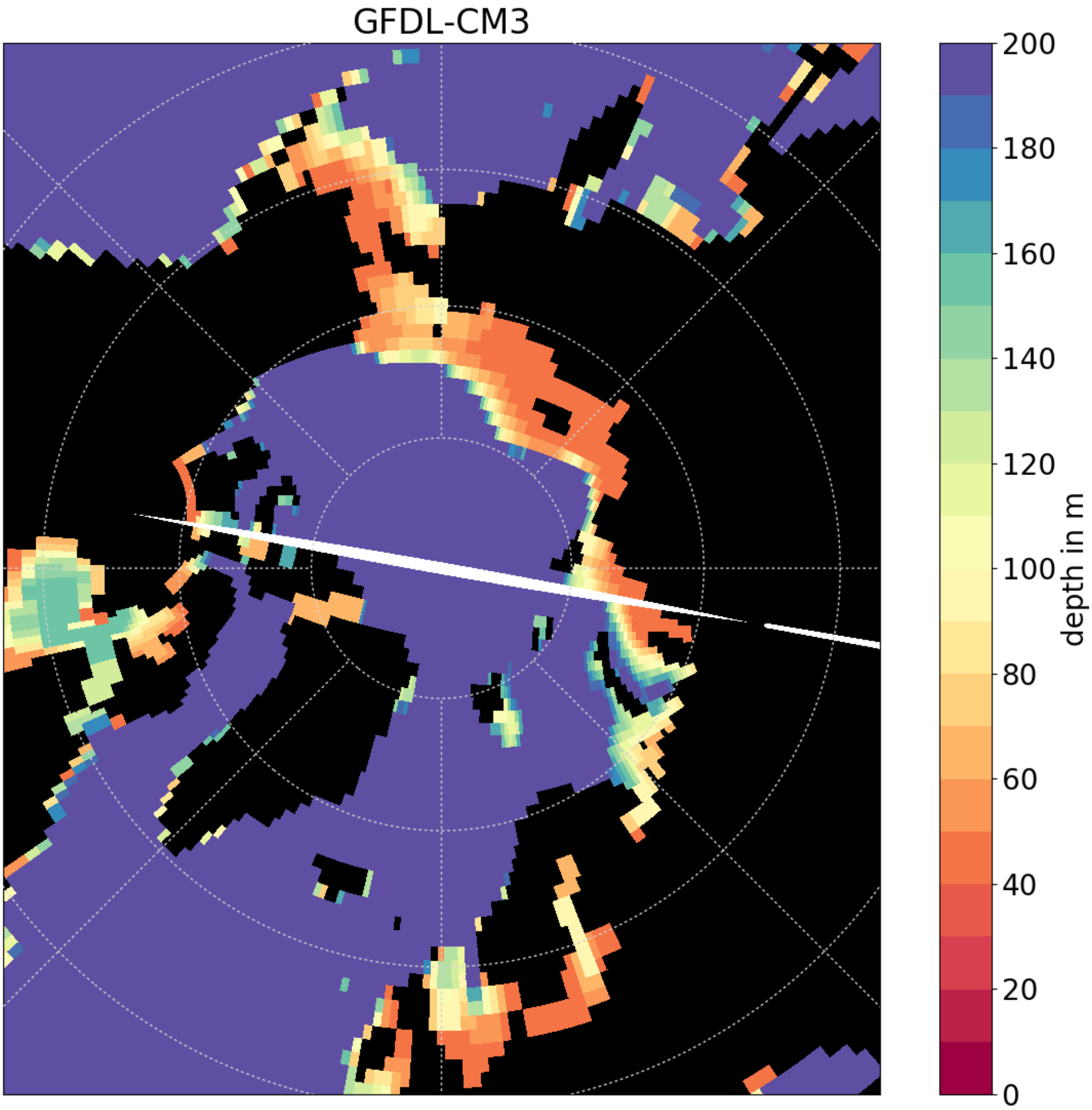

Figure-S3: Ocean depth in m of the CMIP5 model GFDL-CM3. Black denotes land, white indicates undefined area in model grid. The ocean and sea ice model in the GFDL-CM3 has a horizontal resolution of 360x200 grid boxes and uses a B-grid [Griffies et al., 2005].

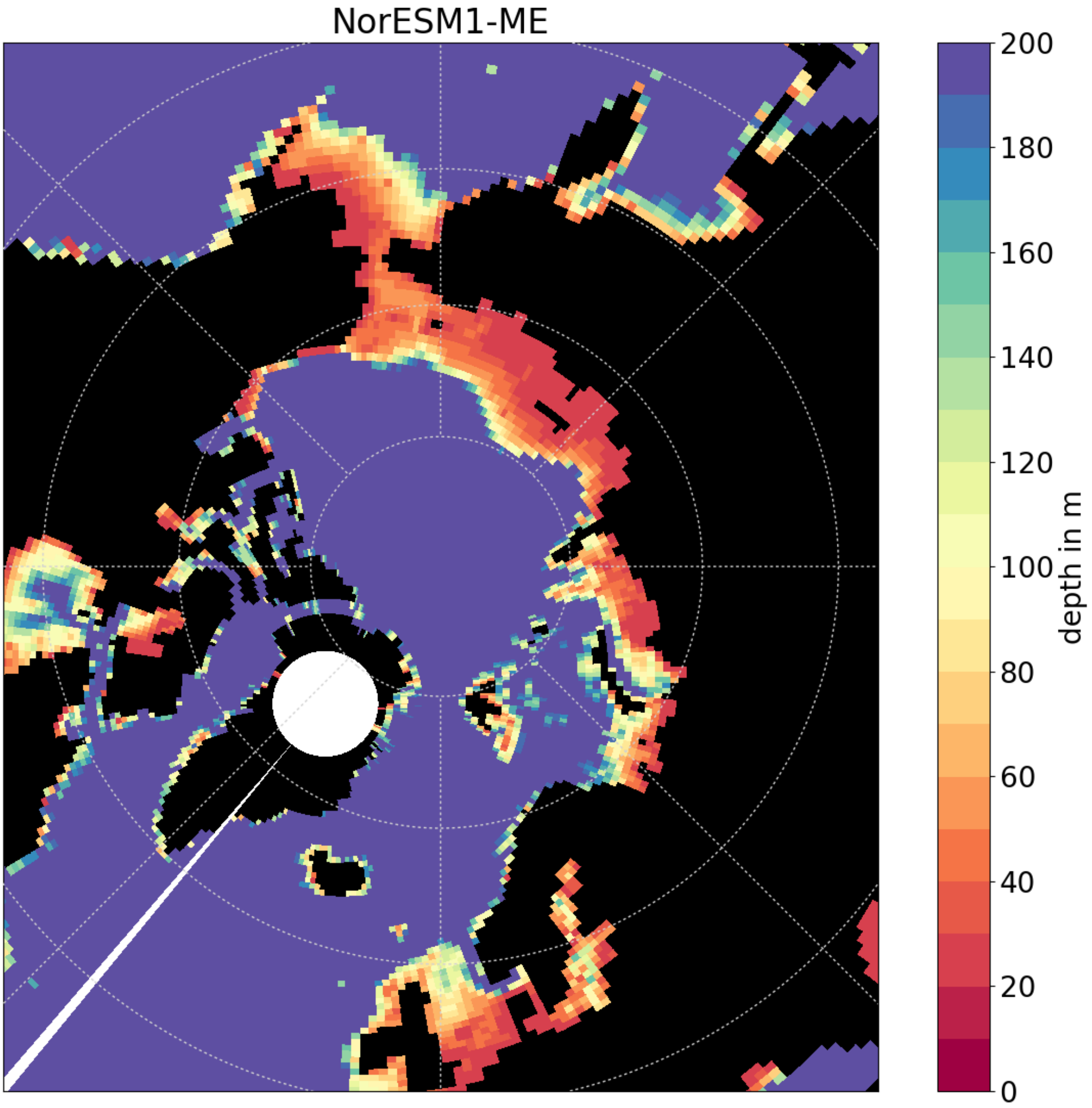

Figure-S4: Ocean depth in m of the CMIP5 model NorESM1-ME. Black denotes land, white indicates undefined area in model grid. The ocean and sea ice model in the NorESM1-ME has a horizontal resolution of 320x384 grid boxes and uses a C-grid .(Bentsen et al., 2013)

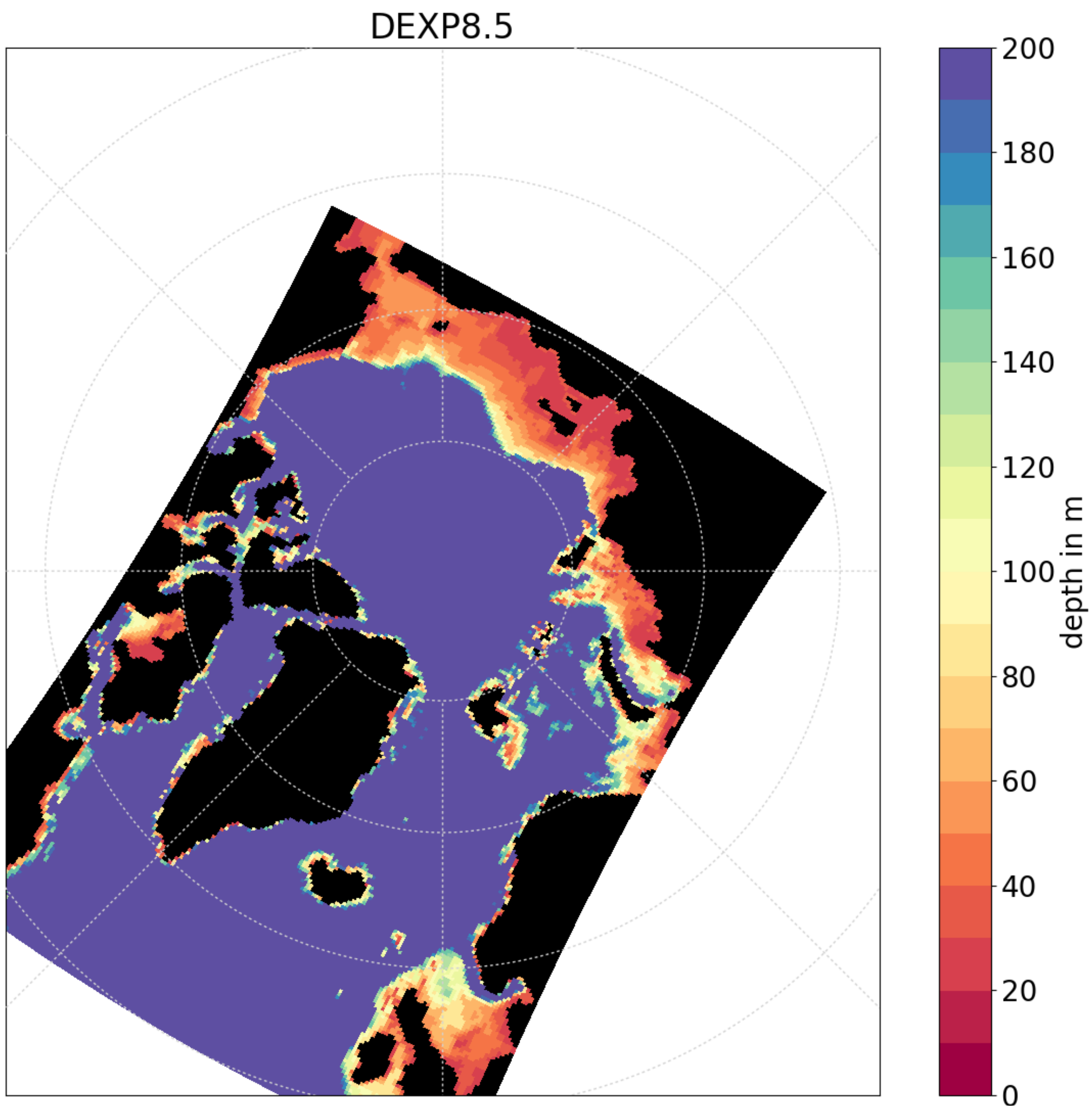

Figure-S5: Ocean depth in m of the downscaled model DEXP8.5. Black denotes land, white indicates undefined area in model grid. The ocean and sea ice model in the DEXP8.5 has a horizontal resolution of 0.25° (243x170 grid boxes) and uses a C-grid.

References

Arakawa, A., and V. R. Lamb (1977), Computational Design of the Basic Dynamical Processes of the UCLA General Circulation Model, in *Methods in computational physics*, edited, pp. 174-267, Academic Press, New York.

Bentsen, M., et al. (2013), The Norwegian Earth System Model, NorESM1-M – Part 1: Description and basic evaluation of the physical climate, *Geoscientific Model Development*, *6*(3), 687-720, doi:10.5194/gmd-6-687-2013.

Griffies, S. M., et al. (2005), Formulation of an ocean model for global climate simulations, *Ocean Science*, *1*(1), 45-79, doi:10.5194/os-1-45-2005.

Hunke, E. C., W. H. Lipscomb, A. K. Turner, N. Jeffery, and S. Elliott (2015), CICE: the Los Alamos Sea Ice Model Documentation and Software User's Manual Version 5.1*Rep.*, Los Alamos National Laboratory.

Jungclaus, J. H., N. Fischer, H. Haak, K. Lohmann, J. Marotzke, D. Matei, U. Mikolajewicz, D. Notz, and J. S. von Storch (2013), Characteristics of the ocean simulations in the Max Planck Institute Ocean Model (MPIOM) the ocean component of the MPI-Earth system model, *Journal of Advances in Modeling Earth Systems*, *5*(2), 422-446, doi:10.1002/jame.20023.

Massonnet, F., T. Fichefet, H. Goosse, C. M. Bitz, G. Philippon-Berthier, M. M. Holland, and P. Y. Barriat (2012), Constraining projections of summer Arctic sea ice, The Cryosphere, 6(6), 1383-1394, doi:10.5194/tc-6-1383-2012.

Overland, J.E. and M. Wang. 2007. Future regional Arctic sea ice declines. Geophysical Research Letters, 34(17), doi: 10.1029/2007gl030808

Overland, J. E., M. Wang, N. A. Bond, J. E. Walsh, V. M. Kattsov, and W. L. Chapman (2011), Considerations in the Selection of Global Climate Models for Regional Climate

Projections: The Arctic as a Case Study*, *Journal of Climate*, *24*(6), 1583-1597, doi:10.1175/2010jcli3462.1.

Petrick, S., K. Riemann-Campe, S. Hoog, C. Growitsch, H. Schwind, R. Gerdes, and K. Rehdanz. 2017. Climate Change, future Arctic Sea ice and the competitiveness of European Arctic offshore oil and gas production on world markets. Ambio. doi:10.1007/s13280-017-0957-z

Riemann-Campe, K., M. Karcher, F. Kauker, and R. Gerdes (2014), D1.51 Results of Arctic ocean-sea ice downscaling runs validated and documented.

Wang, M. Y., and J. E. Overland (2012), A sea ice free summer Arctic within 30 years: An update from CMIP5 models, Geophys Res Lett, 39, L18501, doi:10.1029/2012gl052868.
